# Dengue fever maculopathy: case report and brief review

**DOI:** 10.3205/oc000263

**Published:** 2025-12-05

**Authors:** Aluisio Rosa Gameiro Filho, Willian Gabriel Odorcik, Matheus Henrique Rocha Garcia, Marcelo Brillinger Novello, Daniella Socci da Costa

**Affiliations:** 1 Hospital Regional Doutor Homero de Miranda Gomes (HRSJ), São José, Brazil; 2 Hospital Federal dos Servidores do Estado do Rio de Janeiro (HFSE-RJ), Rio de Janeiro, Brazil

**Keywords:** dengue fever, dengue maculopathy, optical coherence tomography, arboviruses, foveolitis

## Abstract

A 45-year-old woman with a 7-day history of fever and nausea sought assistance at the emergency department complaining of blurred vision in her right eye. She was recently diagnosed with dengue fever. Fundus showed a discrete well-circumscribed, round yellow-orange lesion localized in the foveal region, retinal hemorrhages, macular oedema, soft exudates, and adjacent perivasculitis in the affected eye. Spectral domain optical coherence tomography (OCT) was compatible with dengue maculopathy. She was closely followed, with complete improvement after 1 week. The increasing incidence of dengue makes essential for the ophthalmologist to recognize this rare condition.

## Introduction

Dengue fever is a mosquito-borne infection found in tropical and subtropical regions [1]. It is an important health issue, with transmission occurring in at least 128 countries, causing 390 million infections, and 20,000 fatalities each year [2]. Ocular manifestations can be found in 10% of patients, whereas sight-threatening complications occur in 5–8% of patients [3]. This study reports a rare case of dengue maculopathy in a 45-year-old woman.

## Case description

A 45-year-old woman, otherwise fit and well attended the Emergency Department complaining of blurriness of her right eye (OD), since she woke up on the same day. She denied pain, ocular discharge, or redness. She had a history of joint and muscle pain, which started 8 days before. She went to her general practitioner one day after, when fever and vomit started. At this time, she was evaluated, and the diagnosis of dengue fever (DF) was done and confirmed by serology (NS1 antigen). 

**Figure 1:**
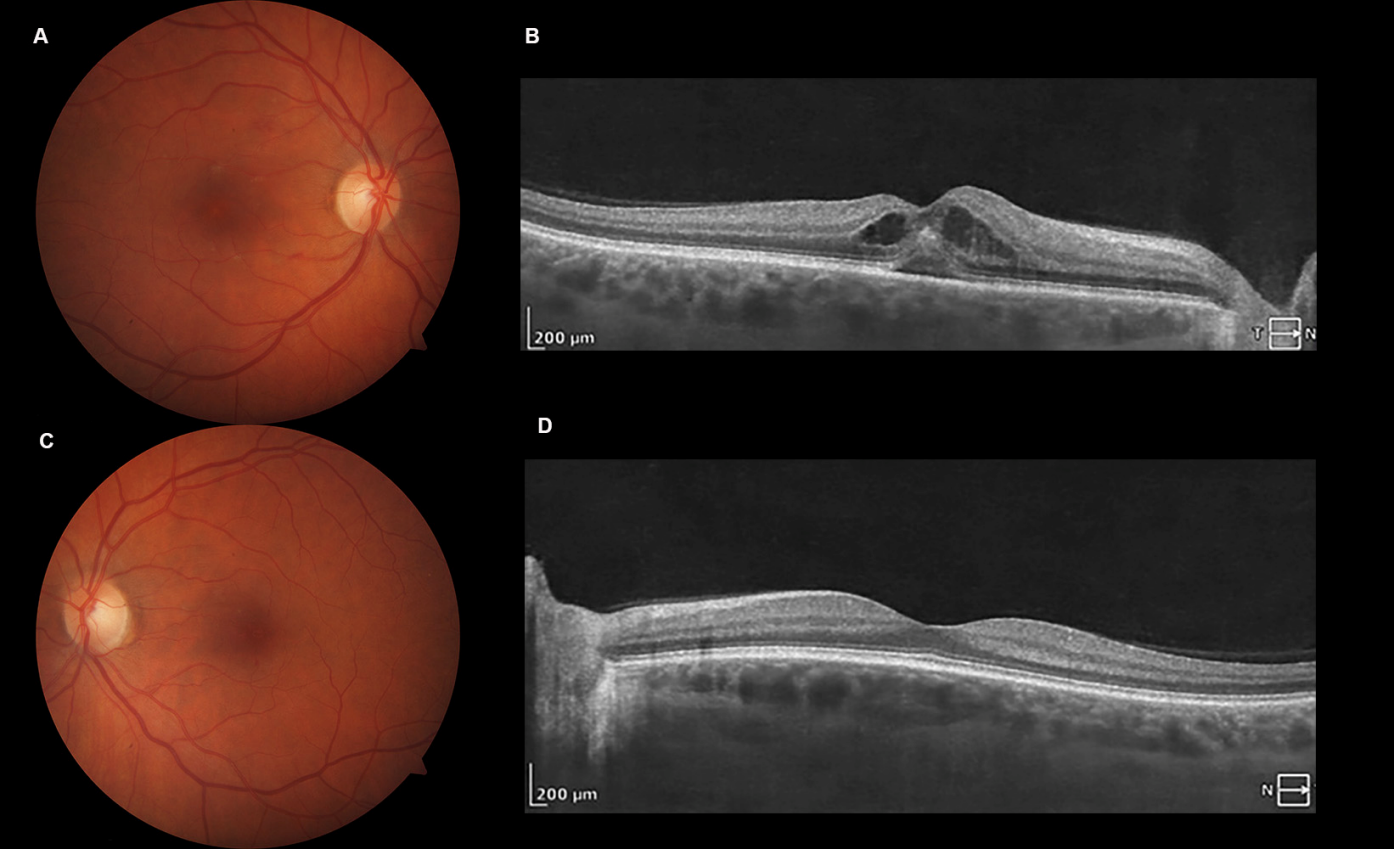
Fundoscopy the first evaluation, showing a discrete well-circumscribed, round yellow-orange lesion localized in the foveal region (foveolitis), retinal hemorrhages, macular oedema, soft exudates, and adjacent perivasculitis in OD (A). Right eye OCT (B) revealing an elevation and disruption of the foveal outer retina photoreceptors and/or retinal pigment epithelium, associated with cystic spaces in the inner retina, and accumulation of SRF. Left eye fundoscopy showing some discrete dot-blot retinal hemorrhages (C). OCT of the same eye was normal (D).

At first ophthalmological evaluation, her best corrected visual acuity (VA) was 20/40 in OD, and 20/20 in the left eye (OS). Biomicroscopy showed no evidence of inflammation, and intraocular pressure (IOP) was normal in both eyes (OU). Fundoscopy revealed a discrete well-circumscribed, round yellow-orange lesion localized in the foveal region, retinal hemorrhages, macular oedema, soft exudates, and adjacent perivasculitis in OD. Left eye showed some discrete dot-blot retinal hemorrhages. Autofluorescence (FAF) showed hypoautofluorescence at the level of retinal hemorrhages, and fluorescein angiography (FA) was unremarkable. Optic coherence tomography (OCT) revealed an elevation and disruption of the foveal outer retina photoreceptors and/or retinal pigment epithelium, associated with cystic spaces in the inner retina, and accumulation of sub-retinal fluid (SRF). Central macular thickness (CMT) was 414 micrometers (µm). OS was normal (Figure 1 (Fig. 1)). OCT angiography (OCT-A) showed deep capillary flow deficit. Facing these symptoms and her medical history, the diagnosis of dengue maculopathy was done, and a complete blood count was requested, revealing hemoglobin of 13, hematocrits of 41% and 54,000 platelets. A complete uveitis panel was also requested, as the institution protocol demands – which includes herpes simplex, varicella zoster, and Epstein-Barr virus, HIV, VDRL, FTA-ABS, COVID-19 and rheumatological tests – with normal results. At this moment, no treatment was prescribed, and we have decided only to follow the patient closely.

**Figure 2:**
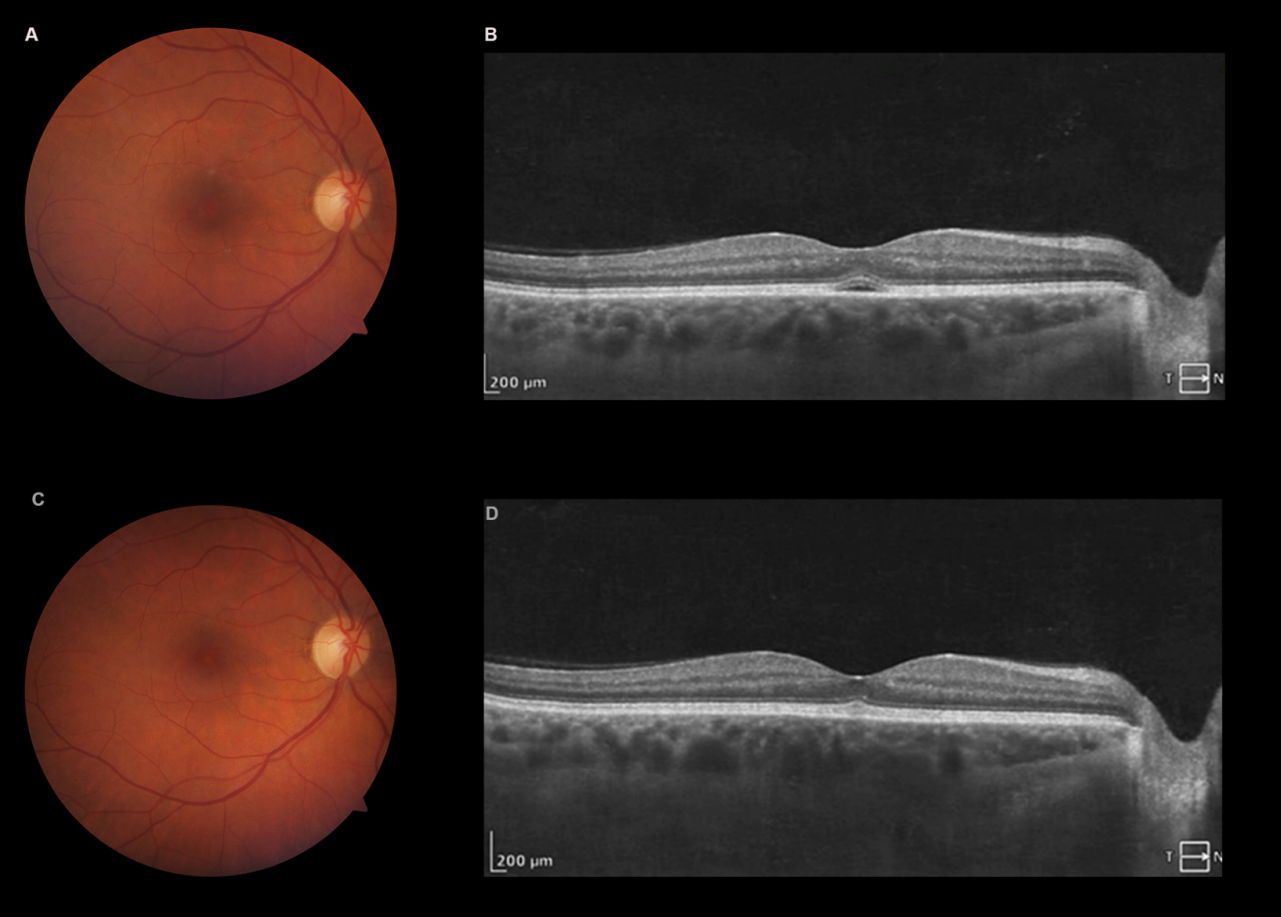
Three days after initial visit, fundoscopy (A) showing improvement, and OCT revealing residual SRF (B). 10 days after first evaluation, fundoscopy (C), and OCT (D) were normal.

She returned 3 days later, referring improvement of her visual acuity (OD – 20/25). A new OCT was performed, showing improvement of the macular findings, with some residual SRF. CMT decreased to 246 µm. One week later, she had no complains, visual acuity in the right eye was 20/20. Fundoscopy and OCT were normal (Figure 2 (Fig. 2)).

## Discussion

Dengue fever is the most common mosquito borne viral disease in humans [4], frequently causing viral epidemics, and imposing a large health care burden. It is estimated that dengue fever threatens about half of the population worldwide, being endemic in 100 countries [5]. According to the World Health Organization (WHO), the incidence of dengue fever has increased from 505.430 notified cases in 2000 to 5.2 million in 2019 [5], which can be explained by urbanization, tourism, and trade [6]. 

This condition is caused by the dengue fever virus (DFV), an RNA virus which belongs to the Flavivirus family. The main vector is Aedes aegypti, a mosquito originated in Africa, which rapidly spread around the globe. It feed almost exclusively on humans in daylight hours, typically rest indoors, and it is frequently found in major cities in the Americas and Asia [4]. Aedes albopictus is also an important vector, mostly in suburban and rural areas [1]. Lastly, Aedes polynesiensis and Aedes scutellaris have been also implicated [6]. 

DFV has four proven serotypes (DENV-1, DENV-2, DENV-3, and DENV-4), and recovery from one infection provides lifelong immunity against that specific serotype, but only partial and transient protection against other serotypes, and sequential infections may increase the risk of more serious systemic – such as dengue hemorrhage fever (DHF) or dengue shock syndrome (DSS) – or ocular disease.

Though 75% of cases of dengue may be asymptomatic [4], patients with symptoms usually present with an abrupt onset of fever 2–7 days after incubation period. Other symptoms include headaches, myalgia, arthralgias, nausea, cutaneous rash, and vomiting. Severe cases can have hypotension, thrombocytopenia, and bleeding. Diagnosis is made by polymerase chain reaction (PCR), virus isolation or detection of viral antigens before fifth day of illness, and serologic tests after 5 days [6].

In the eye, the virus can cause a large broad of manifestation, in several ocular tissues. Manifestations can be either unilateral or bilateral, and time of onset of the symptoms range from 2 days to 5 months, with most occurring within 1 day of the nadir of thrombocytopenia [6]. They include hyposphagma, late-onset anterior uveitis, intermediate uveitis, punctate corneal erosions, corneal ulcer, choroidal effusion, optic disc swelling, optic neuritis, neuromyelitis Optica, panuveitis, and endophthalmitis [7]. Retinal manifestations include vascular occlusions, retinitis, chorioretinitis, neuroretinitis, acute macular neuroretinopathy (AMN) and dengue maculopathy (DVM). 

Patients with DVM are usually have bilateral involvement (80.5%). The commonest symptom is a sudden drop of visual acuity (51.2%), followed by scotomas (34.1%), floaters, micropsia, and metamorphopsia [3]. In the largest case series published, 41.9% patients had visual acuity <20/40 [3]. Additional retinal findings include retinal hemorrhage, vascular sheathing, subretinal dots, retinal pigment epithelium (RPE) mottling, and optic disc edema [8].

Teoh [3] proposed a classification for DVM, based on OCT findings. Type 1 was defined as a diffuse retinal thickening, type 2 as a cystoid macular edema and type 3, as foveolitis, characterized by the disruption of the foveal outer retina photoreceptors and/or retinal pigment epithelium layer, such as in our case. Prognosis is variable, being better in type 1, and worse in type 3. In patients with foveolitis, the VA can be out of proportion to clinical edema, and the amount of retinal thickening.

In most of the patients, fluorescein angiography shows no obvious abnormalities, however, perifoveal leakage, blocked fluorescence and vascular occlusion can be present in some. The most common finding on OCT-A is deep capillary plexus flow deficit, followed by superficial capillary flow deficit [9].

The exact mechanism for ocular manifestations is not yet completely understood, however it is believed that it may be due to a dual mechanism of aggression: ischemia and inflammation. A study demonstrated that DVM is more prevalent in DENV-1 than DENV-2 infection [10]. DENV-1 causes a reactive oxygen species (ROS) attack in a mechanism similar of the one used by SARS-CoV2 [7]. Also, its non-structural protein 1 (NS-1) activates the p38-MAPK pathway, which increases the expression of some endothelial proteins – such as interleukin-10 (IL-10), intercellular adhesion molecule (ICAM), matrix metalloproteinase 2 (MMP-2), transforming growth factor β (TGF-β) and endothelin-1 – leading to hyperpermeability of endothelial cells [10]. Lastly, leukopenia, hypoalbuminemia, and lower levels of C3 and C4 are found in patients with DVM [6].

There is no standard treatment for DVM, as there are no randomized controlled trials to date. As most cases resolve spontaneously over time [6], a conservative approach, with active surveillance may be used in patients with good initial VA. Teoh showed that 62.2% of patient recovered to ≥20/40 at one month [3], and some studies reporting recovering as soon as 3 days after the beginning of symptoms. In other cases, oral or intravenous steroids can be used, as well as immunosuppressants, and intravenous immunoglobulins.

## Conclusion

Dengue fever is the most common arboviruses, and its incidence is increasing over the years, with globalization and international travels, thus the ophthalmologist should be aware of this rare condition, for prompt diagnosis. The symptoms usually start with the beginning of convalescence. Patients in general present with blurred vision, and scotomas. Until the present date, there is no standard treatment. 

## Notes

### Authors’ ORCIDs

- Gameiro Filho AR: 0000-0002-8787-0417
- Odorcik WG: 0009-0004-5529-9561
- Garcia MHR: 0009-0003-4706-6218
- Novello MB: 0009-0006-0167-7322
- da Costa DS: 0000-0002-6457-2118

### Patient consent

Patient has provided written informed consent. 

### Competing interests

The authors declare that they have no competing interests.
